# Functional significance of miR-218 in lung cancer

**DOI:** 10.20517/2394-4722.2025.108

**Published:** 2026-02-13

**Authors:** Divyanshu Aggarwal, Anita Thyagarajan, Ravi P. Sahu

**Affiliations:** Department of Pharmacology and Toxicology, Boonshoft School of Medicine, Wright State University, Dayton, OH 45435, USA.

**Keywords:** Lung cancer, miR-218, cell signaling pathways, tumor suppressors, metastasis, chemotherapy sensitivity, radiation therapy

## Abstract

Lung cancer remains one of the most prevalent and lethal malignancies worldwide, characterized by a poor prognosis and high mortality rates. Although therapeutic strategies targeting oncogenic signaling cascades, such as receptor tyrosine kinases, have shown promising outcomes by regulating cellular functions such as survival and proliferation, their long-term effectiveness is often limited by mechanisms, including tumor resistance, drug toxicity, and adverse events. These challenges underscore the urgent need to identify new molecular targets and develop alternative therapeutic approaches. One promising avenue lies in the exploration of microRNAs (miRs) and their significance in cancer biology. Among them, miR-218 has drawn significant attention for its tumor-suppressive properties. Multiple experimental studies have emphasized the role of miR-218 as a key regulator of cell signaling pathways critical to cancer progression, including proliferation, invasion, metastasis, and apoptosis. Notably, miR-218 has been investigated as both a diagnostic biomarker and a therapeutic target. Importantly, clinical evidence further supports its relevance, showing an inverse correlation between miR-218 expression levels and tumor aggressiveness, reinforcing its translational significance. This review logically consolidates the functional significance, mechanistic insights, and experimental and clinical findings that emphasize the pivotal role of miR-218 in regulating molecular pathways involved in lung cancer growth.

## INTRODUCTION

Lung cancer is the major cause of cancer-related mortalities across the globe, accounting for 18.7% of all cancer deaths in 2022, followed by colorectal cancer (9%)^[1]^. Notably, the cancer statistics estimate that in 2025, new lung cancer cases represent 11% of all cancer cases^[2]^. In terms of gender-based mortality, lung cancer has a mortality rate of 20% in both males and females^[3]^. Histologically, lung cancer is categorized into small-cell lung cancer (SCLC) and non-small-cell lung cancer (NSCLC)^[4]^. NSCLC accounts for 80% of total pulmonary malignancies and is mainly subdivided into three major subtypes, namely, lung adenocarcinoma (LUAD), squamous cell carcinoma (SCC), and large-cell carcinoma (LCC)^[5,6]^, and rare subtypes such as sarcomatoid carcinoma^[7]^. NSCLC involves dysregulation of signaling pathways responsible for cell control, apoptosis, and immune responses, resulting in tumor progression with major mutations observed in the epidermal growth factor receptor (EGFR), Kirsten Rat Sarcoma Viral Oncogene Homolog (KRAS), anaplastic lymphoma kinase (ALK), mesenchymal-epithelial transition Factor (MET), and c-ROS Oncogene 1 (ROS1)^[8,9]^.

Despite significant advancements in the understanding of pathogenic pathways, the exact mechanisms responsible for carcinogenesis and metastasis of NSCLC are still unknown^[10,11]^. In addition, NSCLC is often diagnosed at an advanced stage, which remains one of the major challenges in the treatment of this cancer type^[12–14]^. In fact, the five-year survival rate of NSCLC patients (< 28%) remains relatively unchanged, owing to delayed detection during the early stage of the disease and subsequent progression to an advanced stage before any therapy can be applied^[15–18]^. Although multiple therapeutic strategies are available, including surgical resection for localized tumors, platinum- or taxane-based chemotherapy, and radiotherapy, their efficacy is often reduced when the disease is detected at an advanced stage^[19–21]^. In recent years, the introduction of immune checkpoint inhibitors, as well as targeted therapies directed against oncogenic drivers such as EGFR, has expanded the treatment landscape and provided clinical benefit to selected patient subgroups^[22–25]^. These modalities, together with neoadjuvant and adjuvant approaches, are increasingly being combined to enhance treatment response and improve overall outcomes^[26–28]^.

Lung cancer arises from the uncontrolled proliferation of epithelial cells within the respiratory tract, with tobacco smoking being one of the major risk factors for lung cancer^[29–31]^. Other risk factors include exposure to outdoor air pollution, occupational exposure, radon (especially in non-smokers), family history, and genetic alterations^[8,32–34]^. Thus, understanding the molecular mechanisms underlying its development and progression is critical for improving outcomes^[35]^. To that end, substantial emphasis on ongoing research has been focused on identifying specific molecular players, such as microRNAs (miRs), which regulate gene expression and play a role in carcinogenesis and chemoresistance^[36,37]^.

miRs are single-stranded non-coding RNA molecules comprising 19–25 nucleotides that regulate post-transcriptional gene expression^[38,39]^. The biogenesis of miRs involves the transcription of microRNA (miRNA) coding genes into primary miRNAs (pri-miRNAs), which are then processed to produce precursor miRNAs (pre-miRNAs)^[40]^. The pre-miRNAs are then cleaved to form the miRNA duplex, which involves the integration of various enzymes such as Drosha and RNase Dicer^[40]^. miRs are able to recognize their target messenger RNAs (mRNAs) and by binding to the 3’-untranslated region (3’-UTR) of complementary sequences, they regulate their protein expression^[41]^. This typically results in either translational repression or mRNA cleavage^[42]^. miRs have been found to regulate various key cellular processes, including regulation of the cell cycle^[43,44]^, growth and differentiation^[45]^, migration^[46]^, apoptosis^[47,48]^, and stress response^[49]^.

In cancer, miRNAs can exhibit different functions - namely, tumor-suppressive or tumor-promoting-depending on their target genes, regulatory networks, and ability to regulate cellular processes such as epithelial-mesenchymal transition (EMT)^[50–52]^. Over the last few decades, studies have identified numerous miRNAs that are associated with lung cancer and regulate cellular and chemotherapeutic responses^[53–55]^. Among these, miR-218 has drawn significant attention for its tumor-suppressive properties^[56]^. The focus of this review is to highlight the role and mechanistic insights of miR-218, as well as its impact and implications on the efficacy of therapeutic agents in NSCLC.

## ROLE AND MOLECULAR MECHANISMS OF MIR-218

miR-218 is a highly conserved miRNA encoded by its host genes: Slit Guidance Ligand 2 (SLIT2) and Slit Guidance Ligand 3 (SLIT3) from two distinct loci, miR-218–1 and miR-218–2, which are located on chromosomes 4p15.31 and 5q35.1^[57–59]^. miR-218 is produced from these intronic loci, both of which are frequently deleted or transcriptionally silenced in NSCLC, resulting in decreased expression of miR-218^[58]^. It is predominantly expressed in normal tissues. However, in cancerous tissues, miR-218 is frequently downregulated, contributing to the dysregulation of its target genes and associated pathways^[60–63]^. Beyond canonical transcription and processing, miR-218 is also regulated through non-canonical mechanisms involving its genomic context and long non-coding RNA (lncRNA) networks. Structural loss or epigenetic silencing of SLIT2/SLIT3 decreases intronic miR-218 transcription, while lncRNAs such as CCAT1 (colon cancer-associated transcript 1) act as miR-218 sponges, thereby derepressing B lymphoma Mo-MLV insertion region 1 homolog (BMI-1) and promoting tumor growth and drug resistance in lung cancer^[64,65]^.

Experimental evidence suggests that miR-218 functions as a tumor suppressor in various malignancies, such as breast cancer^[66]^, colorectal cancer^[67]^, retinoblastoma^[68]^, cervical cancer^[69]^, thyroid cancer^[70]^ and lung cancer^[71]^. In lung cancer, miR-218 has been found to regulate several key oncogenic pathways, including the phosphoinositide 3-kinase/protein kinase B (PI3K/AKT) pathway, the interleukin-6/Janus kinase/signal transducer and activator of transcription 3 (IL-6/JAK/STAT3) pathway, and IκB (inhibitor of κB), a key component of the nuclear factor-kappa B (NF-κB) signaling pathway, all of which play critical roles in tumor progression^[56,72]^. Alongside that, it directly targets genes such as EGFR, BMI-1, and B-cell lymphoma (Bcl-2), which are critical in tumor development and metastasis^[72,73]^. The loss of miR-218 expression in lung cancer correlates with aggressive tumor phenotypes, tumor, node, metastasis (TNM) stages, and poor patient prognosis^[73]^. Below, we logically discuss the *in vitro* studies, *in vivo* studies, clinical evidence and bioinformatics evidence exploring the functional significance of miR-218 alone or its combination with therapeutic agents in lung cancer models, with major focus on NSCLC.

## IMPLICATIONS OF MIR-218 IN LUNG CANCER

### Evidence from the *in vitro* studies

**Figure 1.**
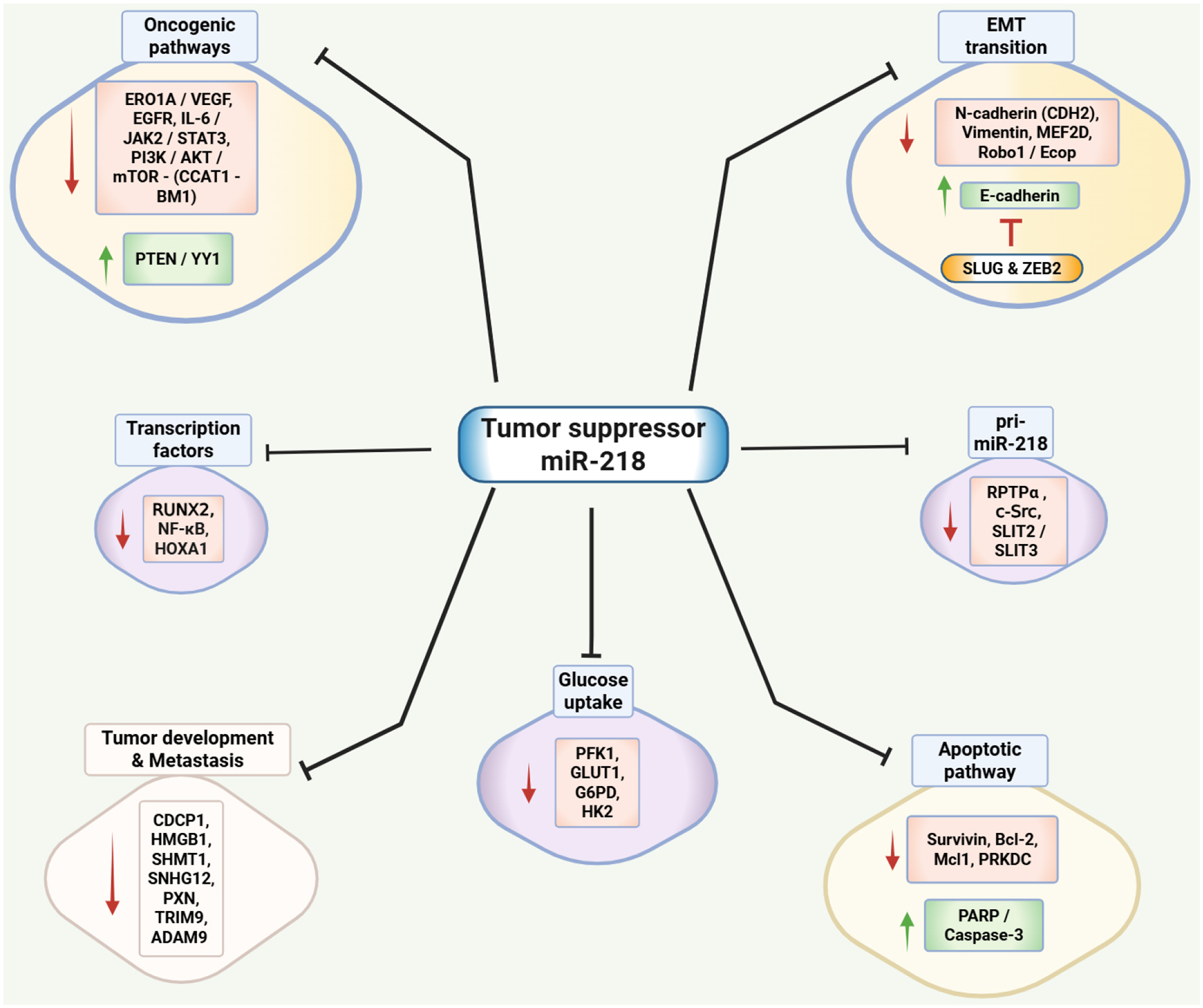
Schematic representation of miR-218 mechanisms of action. miR-218 targets multiple oncogenic cascades, transcription factors, apoptotic pathways, and cellular properties such as tumor development and metastasis, glucose uptake, and EMT, as well as reduces its own synthesis by inhibiting pri-miR-218. ERO1A: Endoplasmic reticulum oxidoreductase 1 alpha; VEGF: vascular endothelial growth factor; PI3K/AKT/mTOR: phosphoinositide 3-Kinase/protein kinase B/mammalian target of rapamycin; IL-6/JAK/STAT3: interleukin-6/Janus kinase 2/signal transducer and activator of transcription 3; CCAT1: colon cancer-associated transcript 1; BMI-1: B lymphoma Mo-MLV insertion region 1 homolog; PTEN/YY1: phosphatase and tensin homolog/Yin Yang 1; PARP: poly-ADP ribose polymerase; Bcl-2: B-cell lymphoma; Mcl1: myeloid cell leukemia sequence 1; HOXA1: homeobox A1; RUNX2: runt-related transcription factor 2; PRKDC: protein kinase, DNA-activated, catalytic subunit; RPTPα: receptor protein tyrosine phosphatase alpha; SLIT2/SLIT3: Slit Guidance Ligand 2/3; TRIM9: tripartite motif 9; SNHG12: small nucleolar RNA host gene 12; SHMT1: serine hydroxymethyltransferase 1; CDCP1: CUB-domain-containing protein 1; ADAM9: disintegrin and metalloproteinase domain 9; HMGB1: high mobility group box-1; PXN: paxillin; EGFR: epidermal growth factor receptor; NF-κB: Nuclear factor kappa-light-chain-enhancer of activated B cells; PFK1: phosphofructokinase 1; GLUT1: glucose transporter 1; G6PD: glucose-6-phosphate dehydrogenase; HK2: hexokinase 2; MEF2D: myocyte enhancer factor 2D; Robo1: roundabout homolog 1; Ecop: EGFR-coamplified and -overexpressed protein; CDH2: N(Neural)-cadherin; ZEB2: zinc finger E-box-binding homeobox 2; EMT: epithelial-mesenchymal transition. Created in BioRender Thyagarajan A (2025).

Several studies have utilized *in vitro* culture models to define the mechanism and functional significance of miR-218 in NSCLC. One of the first studies to correlate miR-218 with NSCLC was conducted by Wu *et al*.^[74]^. The study focused on determining the impact of miR-218 on the proliferative and invasion ability of NSCLC cells. The researchers initially used a wide panel of lung cancer cell lines, including A549, H1299, Ch27, H460, Calu-1, H661, CL1–0, and CL1–5. However, upon analyzing the miR-218 expression levels in these cell lines, the authors performed further studies primarily using CL1–0 and CL1–5 cell lines. The data demonstrated that overexpression of miR-218 inhibited the growth and invasive potential of lung cancer cells, while its inhibition enhanced these aggressive phenotypes. Additionally, they also discovered an inverse correlation in miR-218 expression and paxillin (PXN) mRNA and protein levels, which are associated with poor overall survival (OS) and relapse-free survival (RFS). The comparative summary of the *in vitro* studies is given in Table 1, and the schematic representation of miR-218 targets is shown in Figure 1.

Wang *et al*. conducted a study focusing on the effect of miR-218 in restricting cell proliferation and metastasis by targeting TRIM9, a member of the tripartite motif (TRIM) family^[80]^. This protein has been associated with tumorigenesis in several cancers^[92,93]^. The data demonstrated that while miR-218–5p was significantly downregulated, TRIM9 was upregulated in 95D and H1299 NSCLC cell lines^[80]^. This suggested a negative correlation between the expression levels of miR-218–5p and TRIM9, which was confirmed using the luciferase reporter assay. In addition, overexpression of miR-218–5p led to a significant reduction in cell proliferation, migration, and invasion in NSCLC cells. The same effects were documented following the knockdown of TRIM9, suggesting that TRIM9 acts as an oncogene in NSCLC^[80]^. Furthermore, overexpression of TRIM9 reversed the suppressive effects of miR-218–5p, reinforcing the notion that TRIM9 is a downstream target of miR-218–5p^[80]^. The study thus revealed that since TRIM9 is associated with poor prognosis in lung cancer, targeting the miR-218–5p/TRIM9 axis could offer a novel therapeutic strategy for NSCLC.

As mutations in the *EGFR* gene contribute to nearly one-third of all NSCLC cases, especially in regions such as Asia^[94]^. This makes EGFR a potential target to determine the mechanistic insights and explore its impact on the treatment strategies in NSCLC. To that end, Zhu *et al*. conducted a study to explore correlation between miR-218 and EGFR expression levels using the LUAD A549 and H1975 cell lines^[73]^. They found that miR-218 directly targets EGFR protein in NSCLC cells, with miR-218 overexpression leading to reduced cell proliferation and migration. This correlation with EGFR provides the rationale for exploring miR-218 application in the treatment strategies of NSCLC.

EMT is a biological process in which epithelial cells lose their cell-cell adhesion and epithelial markers, and acquire mesenchymal characteristics such as increased motility and invasiveness, enabling metastasis^[95]^. Since EMT is a key characteristic of NSCLC, Zhang *et al*. investigated the role of miR-218 in NSCLC cell migration using the A549 and H1299 cell lines^[81]^. The study showed that overexpression of miR-218 using mimetics resulted in the reduction of cell migration and invasion. Furthermore, the study also identified high-mobility group box 1 (HMGB1) as a direct target of miR-218. Given that high HMGB1 levels have been associated with poor clinical outcomes and metastasis in lung cancer, exploration of the mechanisms involved in its regulation such as miR-218 has potential significance^[96]^. A similar finding was reported by Sher *et al*., using BM7 cell model (BM7 cell line was a brain-metastatic clone derived from a high metastatic subline F4, which had higher invasion capability than its parental cell line), where miR-218 was found to target N-cadherin (CDH2), which is a mesenchymal marker of the EMT process^[82]^. The studies primarily focused on a disintegrin and metalloprotease 9 (ADAM9), which was found to upregulate N-cadherin via suppressing miR-218 level, thus promoting metastasis in NSCLC.

Moreover, to evaluate the significance of miR-218 in EMT and metastasis, Shi *et al*. conducted a study using EMT markers such as E-cadherin and vimentin^[83]^. They selected a panel of lung cancer cell lines - A549, H1299, PC9 and SPCA-1, and first assessed the expression levels of miR-218, and then chose H1299 and A549 cells for further experiments. During EMT, E-cadherin expression decreases, leading to loss of cell-cell adhesion and epithelial characteristics, while Vimentin increases, promoting cell motility and structural reorganization^[97]^. Interestingly, the study found that miR-218 overexpression reversed this pattern, increasing E-cadherin and decreasing Vimentin. Furthermore, miR-218 transfection was able to induce morphological changes in NSCLC cells. Additionally, the transcription factor, Slug and zinc finger E-box-binding homeobox 2 (ZEB2), which typically represses E-cadherin, were identified as direct targets of miR-218. This study holds great significance as it demonstrated the potential of miR-218 to prevent metastasis in lung cancer.

Furthermore, a study conducted by Li *et al*. demonstrated that miR-218 exerts its action on inhibiting EMT by targeting several key components such as Roundabout homolog 1 (Robo1) and EGFR-coamplified and -overexpressed protein (Ecop)^[79]^. Robo1 is an axon guidance receptor gene that has been shown to enhance migratory capacity in breast cancer^[98]^. Ecop is a novel oncogenic protein that directly regulates NF-κB transcriptional activity, thereby promoting cancer cell survival through suppression of apoptosis^[99]^. The study provided compelling evidence that the expression levels of miR-218 are negatively correlated with those of Robo1 and Ecop in A549, H2228, H1975, HCC4006, H23, Calu-3, H1435 and H1793 LUAD cell lines. Furthermore, miR-218 repressed cancer cell invasion and migration by inhibiting Robo1 and Ecop expression, reinforcing the notion that miR-218 plays a critical role in suppressing tumor progression. In fact, similar findings were reported by Chen *et al*., where the researchers experimented only on Robo1 expression and found that miR-218 transfection significantly reduces the expression of Robo1, affecting cell migration and invasion in A549 cells^[84]^.

Along similar lines, Wang *et al*. conducted a study to delve further into the mechanisms of the EMT pathway and determined the effects of miR-218 in the same context, using A549 and H1299 NSCLC cell lines^[100]^. They identified small nucleolar RNA host gene 12 (SNHG12), a lncRNA with functions in tumor progression and treatment resistance of NSCLC^[101]^, as a key regulator of miR-218 in EMT. The authors found that SNHG12 was overexpressed in NSCLC tissues with significant correlations with advanced tumor stages and poor OS rates and that silencing SNHG12 led to a marked decrease in cell viability, proliferation, migration, and invasion. They further found that SNHG12 acts as a molecular sponge for miR-218, sequestering and preventing its regulatory effects on target genes. However, the knockdown of SNHG12 resulted in the upregulation of miR-218 followed by downregulation of the Slug/ZEB2 signaling pathway known to regulate EMT and metastasis.

A study by Song *et al*. focused on the miR-218 effect on NSCLC via myocyte-specific enhancer factor 2D (MEF2D) regulation using A549 lung carcinoma cell line^[86]^. MEF2D functions as an oncogene in lung cancer by inducing EMT and promoting tumor cell proliferation and motility^[102]^. The researchers found that mimic transfection of miR-218 directly targets the MEF2D expression resulting in decreased cell proliferation, survival, and invasion. Additionally, the use of miR-218 inhibitors had the opposite effect, enhancing the malignant traits, suggesting an inverse correlation between miR-218 and MEF2D expression levels.

The role of miR-218 in cancer metastasis was further explored where ADAM9 and CUB domain-containing protein 1 (CDCP1) were used as the main targets of the study^[76]^. ADAM9 is known for promoting metastasis and CDCP1 is a transmembrane glycoprotein that plays a significant role as a driver of oncogenic signaling in tumor progression and metastasis in various cancers^[103–105]^. In the study, the researchers found that miR-218 directly targets the 3’-UTR of CDCP1 mRNA, which resulted in the inhibition of the migration ability of A549 and F4 lung cancer cells. Furthermore, it was found that ADAM9 enhances CDCP1 expression by suppressing miR-218 levels, thus creating a feedback loop where ADAM9 promotes CDCP1 expression, which in turn facilitates cancer cell migration and metastasis. Similarly, Zeng *et al*. also identified CDCP1 as a direct target of miR-218 using the A549 cell line^[77]^. The study demonstrated that overexpression of miR-218 resulted in a reduction in CDCP1 expression, suggesting that miR-218 exerts its tumor-suppressive effects in NSCLC by down-regulating CDCP1 expression.

A study conducted by Zarogoulidis *et al*. found that miR-218, when upregulated, resulted in reduced cell viability and induced apoptosis in A549 and H1975 lung cancer cell lines^[75]^. The authors explored the effects of miR-205 and miR-218 on apoptosis and their roles in the chemoresistance of carboplatin, a platinum-based medication used to treat lung cancer. The researchers found that while miR-205 promotes chemoresistance, miR-218 upregulation inhibited that by reducing the expression of specific anti-apoptotic proteins, namely survivin and Myeloid cell leukemia 1 (Mcl-1). Mcl-1 is known to promote cell survival by inhibiting apoptosis, while survivin plays a critical role in apoptosis regulation^[106]^. Furthermore, miR-218 also promoted the expression of apoptotic proteins, including poly-ADP ribose polymerase (PARP), Bax and Caspase-3 resulting in the induction of apoptosis. Along similar lines, Zhao *et al*. found that downregulation of miR-218 increases cell proliferation and decreases apoptosis^[64]^. They conducted a study focused on the significance of CCAT1/mir-218 on cell proliferation and apoptosis in NSCLC using A549 and H1975 cell lines and patient tissues. The authors found that patients with high expression levels of CCAT1 had a lower survival rate and poor prognosis than those with lower expression. In NSCLC cells, CCAT1 knockdown led to a decrease in cell proliferation and an increase in apoptosis. However, this effect was reversed when miR-218 was downregulated. The authors also identified a new target of miR-218, BMI-1, which is a member of the polycomb group proteins (PcG) and has been shown to be involved in tumorigenesis^[107]^. BMI-1 was found to be downregulated with CCAT1 knockdown and targeted by miR-218.

Chen *et al*. conducted a study to examine the effects of miR-218 in NSCLC. However, unlike other studies, they used an adenocarcinoma cell line, XWLC-05 (Xuanwei lung adenocarcinoma cell line-05), named after Xuanwei county in China^[72]^. The researchers found that miR-218 is significantly downregulated in lung cancer cells compared to normal lung epithelial cells. In addition, overexpression of miR-218 significantly inhibited the proliferation, invasion, and migration in XWLC cells, while also inducing apoptosis. Furthermore, miR-218 overexpression led to a significant accumulation of cells in the G2 phase of the cell cycle, suggesting that it induces G2/M phase arrest. Additionally, miR-218 overexpression downregulated the levels of Bcl-2 and BMI-1, which are known to promote cell survival and proliferation. In addition, increased expressions of phosphatase and tensin homolog (PTEN) and Yin Yang 1 (YY1) were noticed by miR-218 overexpression, which is associated with tumor suppression and cell cycle regulation. Bcl-2 is an anti-apoptotic protein and PTEN is a tumor suppressor and a negative regulator of the PI3K/AKT/mammalian target of rapamycin (mTOR) oncogenic signaling pathway^[108]^. This study provided insights into the possible roles of miR-218 in NSCLC treatment. Chen *et al*. explored the effect of miR-218 on serine hydroxymethyltransferase 1 (SHMT1), a cytoplasmic enzyme involved in one-carbon metabolism in NSCLC cells^[87]^. SHMT1 is known to play a significant role in cancers due to its involvement in cellular metabolism and DNA synthesis^[109]^. Treatment with recombinant miR-218 resulted in a significant decreased level of the SHMT1 protein in both A549 and H1975 NSCLC cell lines, which led to disruptions in the folate one-carbon metabolism, primarily in the incorporation of the methylene bridge from serine into the folate cycle^[87]^. Furthermore, the downregulation of SHMT1 was also associated with reduced cell viability and proliferation among NSCLC cell lines^[87]^. This interaction between miR-218–5p and SHMT1 elucidates a mechanism by which miR-218–5p functions as an antimetabolite, disrupting key metabolic pathways that NSCLC cells rely on for growth and survival^[87]^.

As vascular endothelial growth factor (VEGF) is a key mediator of angiogenesis, which plays a critical role in NSCLC by supplying oxygen and nutrients to rapidly growing tumors, facilitating tumor progression, and metastasis^[110]^. Heparan sulfate D-glucosamine 3-O-sulfotransferase 3B1 (HS3ST3B1) is an enzyme involved in the sulfation of heparan sulfate which plays a role in enhancing VEGF signaling in leukemias and NSCLC^[111]^. A study by Zhang *et al*. found that HS3ST3B1 is upregulated in NSCLC tissues compared to matched normal tissues, with higher mesenchymal phenotype expression in CALU6, H460, H1975, A549, HCC827, and H358 NSCLC cell lines^[85]^. While its knockdown reversed the EMT-induced responses, it was found that transfection with miR-218 mimics also led to decreased protein levels of HS3ST3B1 in NSCLC cell lines, which inhibited the EMT process. Along similar lines, Chen *et al*. determined the effect of miR-218 on VEGF secretion, focusing on endoplasmic reticulum oxidoreductase 1 alpha (ERO1A), which was identified as a potential target of miR-218 using bioinformatics software^[88]^. ERO1A is an oxidoreductase in the endoplasmic reticulum, known to promote angiogenesis by enhancing the secretion of VEGF. Using a luciferase assay, the authors confirmed that ERO1A was a direct target of miR-218 in PC-9 and A549 cell lines. In addition, while high expression of ERO1A in LUAD correlates with aggressive tumor behavior, miR-218–5p was found to exert its tumor-suppressive effects by targeting ERO1A. These findings suggest the possibility of a regulatory axis where miR-218–5p inhibits LUAD progression by downregulating ERO1A.

Moreover, miR-218’s tumor suppressor activity was found to be mediated via its ability to target various other pathways in NSCLC. One of those is the IL-6/JAK/STAT3 signaling pathway, which is responsible for promoting cancer cell proliferation, angiogenesis, and resistance to apoptosis^[112]^. Yang *et al*. found that miR-218 directly targets the IL-6/Janus kinase 3 (JAK3)/STAT3 pathway in A549 and H1975 cell lines, which resulted in reduced cell proliferation and invasive behavior of cancer cells^[56]^. Additionally, the authors also compared the miR-218 expression with disease prognosis and found that decreased levels of miR-218 were associated with poorer prognosis in NSCLC patients. Lai *et al*. also elucidated the molecular mechanisms of miR-218, though primarily focusing on the receptor-type protein tyrosine phosphatase α (RPTPα)-c-Src signaling pathway using A549 cells as an NSCLC model^[78]^. In general, the RPTPα activates c-Src through dephosphorylation of its inhibitory tyrosine residue (Tyr527), leading to increased c-Src kinase activity. In lung SCC, elevated RPTPα expression correlated with poor prognosis and cell cycle dysregulation, as RPTPα overexpression was shown to accelerate the G1/S transition by upregulating cyclin D3 and cyclin-dependent kinase 4 (CDK4), resulting in increased cancer cell proliferation^[113]^. In addition, the researchers demonstrated that miR-218 directly targets RPTPα mRNA, leading to decreased activation of c-Src as evidenced by lower levels of phosphorylated c-Src. This indicates a negative feedback loop where miR-218 inhibits RPTPα, which in turn reduces c-Src activity. Moreover, c-Src was found to suppress miR-218 expression by downregulating its host genes, SLIT2 and SLIT3, which encode pri-miR-218. This indicates a feedback loop where c-Src not only acts downstream of RPTPα but also negatively regulates miR-218, promoting a pro-tumorigenic environment. Overall, this study had significant implications as the identification of miR-218 as a regulator of the RPTPα-c-Src signaling pathway opens new avenues for targeted therapies that could improve patient outcomes.

Glucose metabolism is essential for cancer progression because it provides the substrates required for tumor growth and metastasis^[114]^. Tian *et al*. studied the effects of miR-218 on glucose consumption, using the NCI-H23 and A549 human NSCLC cell lines and miR-218 mimics^[89]^. The authors found that miR-218 overexpression significantly reduced glucose consumption and led to a decrease in glucose uptake and lactate production in NSCLC cells. In addition, the ratio of Nicotinamide Adenine Dinucleotide Phosphate (reduced form) (NADPH) to Nicotinamide Adenine Dinucleotide Phosphate (oxidized form) (NADP^+^), a measure of pentose phosphate pathway (PPP) activity, was also reduced in cells expressing higher levels of miR-218. Furthermore, the authors determined the expression levels of glucose transporter 1 (GLUT1), hexokinase 2 (HK2), phosphofructokinase 1 (PFK1) and glucose-6-phosphate dehydrogenase (G6PD), and identified GLUT1 as a direct target of miR-218 in NSCLC cells. Further analysis revealed that miR-218 can inhibit glucose uptake by downregulating GLUT1 expression. Moreover, miR-218 significantly decreased the phosphorylation of NF-κB p65 in NSCLC cells, suggesting that the miR-218-induced inhibition of glucose metabolism was mediated by the NF-κB signaling pathway.

Chemotherapeutic agents represent viable treatment options for human cancers and miRs are being evaluated for their impact on drug resistance. To that end, Xie *et al*. conducted a study to elucidate the function of miR-218 on cisplatin chemoresistance using parental A549 and A549/DDP (cisplatin-resistant) cell lines^[90]^. The researchers found that expression levels of miR-218 were significantly downregulated in cisplatin-resistant A549/DDP NSCLC cells compared to their parental A549 cells. Restoring the miR-218 levels led to an increased sensitivity of cisplatin-resistant cells to cisplatin. Furthermore, the study identified Runt-related transcription factor 2 (RUNX2) as a direct target of miR-218. RUNX2 is a transcription factor known to be involved in various cellular processes, including proliferation, differentiation, and apoptosis in various cancer models^[115]^. The study demonstrated that the restoration of miR-218 levels may be a potential therapeutic strategy for reversing the chemoresistance to cisplatin.

Given the intriguing role of miR-218 in the regulation of NSCLC growth, Jin *et al*. determined the effect of miR-218 and lncRNA CCAT1 in drug resistance in NSCLC focusing on a tyrosine kinase inhibitor, gefitinib^[65]^. To that end, they used HCC827 and PC9 cell lines and generated gefitinib-resistant cell lines, HCC827GR and PC9GR. The authors found that CCAT1 expression was higher in gefitinib-resistant NSCLC cell lines compared to gefitinib-sensitive counterparts and that its knockdown enhanced gefitinib sensitivity in NSCLC cells. Furthermore, the authors found that CCAT1 acts as a molecular sponge of miR-218 and reduces its expression levels. The study further identified homeobox A1 (HOXA1) as a direct target of miR-218. HOXA1 is upregulated in NSCLC and promotes tumorigenesis^[116]^. The study suggested that CCAT1 reduces the expression of miR-218, enabling the upregulation of HOXA1, which promotes chemoresistance. This study established that restoring miR-218 levels could be a promising therapeutic strategy to overcome resistance to gefitinib and improve treatment outcomes for patients with NSCLC.

Moreover, radiation therapy has been used for decades to manage NSCLC; however, patients often develop resistance to this therapy^[117]^. To that end, Chen *et al*. explored whether miR-218 could be used to enhance the sensitivity of radiation-resistant lung carcinoma cells^[91]^. For that, A549 and H1299 cell lines were irradiated with a 2 Gy daily fraction size administered 30 times for a total dose of 60 Gy. The authors found that overexpression of miR-218–5p in radiation-resistant NSCLC cell lines led to a significant reduction in cell viability and increased apoptosis following X-ray irradiation. These findings indicate that miR-218–5p enhances the sensitivity of these NSCLC cell lines to radiation therapy. Furthermore, the study identified protein kinase, DNA-activated, catalytic subunit (PRKDC) as a direct target of miR-218–5p. The inhibition of PRKDC expression was found to be associated with increased DNA damage^[118]^. This suggests that miR-218–5p may sensitize cancer cells to radiation by impairing their ability to repair DNA damage. Overall, these findings suggest that by modulating miR-218–5p levels, it may be possible to enhance the efficacy of radiotherapy and improve patient outcomes in NSCLC.

Although most studies have characterized miR-218 as a tumor-suppressive miR in lung cancer, a contrasting report by Yang *et al*. demonstrated that miR-218 can exert a pro-tumorigenic effect within the immune microenvironment^[119]^. The authors investigated the immunomodulatory role of miR-218–5p in LUAD, focusing on its impact on the cytotoxic activity of natural killer (NK) cells through targeting SHMT1. They found that miR-218–5p was significantly upregulated in NK cells isolated from LUAD patients, whereas SHMT1 expression was markedly reduced, suggesting an inverse relationship between the two molecules. This negative regulatory interaction was confirmed via luciferase reporter assays, which validated SHMT1 as a direct downstream target of miR-218–5p^[119]^. Functionally, overexpression of miR-218–5p in interleukin 2 (IL-2)-activated NK-92 cells led to a pronounced reduction in Interferon-gamma (IFN-γ) and tumor necrosis factor-alpha (TNF-α) secretion, accompanied by significant suppression of NK-cell-mediated cytotoxicity against A549 LUAD cells. Conversely, knockdown of miR-218–5p or overexpression of SHMT1 restored cytokine production and enhanced NK-cell killing capacity. Taken together, these findings indicate that, unlike its tumor-suppressive effects reported in lung cancer epithelial cells, miR-218–5p exerts a pro-tumorigenic, immune-evasive role within NK cells mediated by downregulation of SHMT1 and impaired NK-cell cytotoxicity^[119]^.

While the majority of studies discussed in this section consistently demonstrate a tumor-suppressive role for miR-218 in lung cancer cells - mediating inhibition of proliferation, migration, invasion, EMT, and oncogenic signaling pathways, the findings by Yang *et al*. revealed a critical context-dependent divergence^[119]^. Unlike the epithelial cell-intrinsic effects described throughout the preceding studies, miR-218–5p can exert a pro-tumorigenic influence in immune cells by impairing anti-tumor immunity. This discrepancy underscores the cell-type specificity of miR function and emphasizes that the effects of miR-218 are highly dependent on biological context. Therefore, therapeutic strategies targeting miR-218 must account for these dual roles to avoid inadvertently compromising immune-mediated tumor control.

### Evidence from the *in vivo* studies

While *in vitro* studies provide mechanistic insight into miR-218-mediated signaling, many investigations have utilized *in vivo* mouse models to establish the functional significance of miR-218 in NSCLC. To explore the therapeutic potential of miR-218 in modulating lung tumor metastasis, Chiu *et al*. utilized severe combined immunodeficient (SCID) mice and injected intracardially with human lung cancer cells (Bm7brmx2) that had inducible miR-218 expression^[76]^. Mice were treated with doxycycline to induce miR-218 expression. Results showed that induction of miR-218 significantly reduced tumor metastasis to the brain, as evidenced by bioluminescent imaging and histological analysis. In addition, survival analysis demonstrated that mice receiving miR-218 treatment had a significantly longer survival time than the control group, highlighting the potential of miR-218 as a therapeutic agent. The study highlighted the potential of miR-218 as a candidate for therapeutic intervention to combat lung cancer metastasis, which remains a major challenge in oncology. Table 2 lists the summaries of the *in vivo* studies in chronological order of their publication.

Along similar lines, Lai *et al*. conducted *in vivo* studies to assess the role of miR-218 in inhibiting NSCLC tumor growth using BALB/c nude mice^[78]^. To that end, stable A549 cell lines were engineered to overexpress miR-218, while cells without miR-218 expression served as controls, and were then injected subcutaneously into the flanks of male nude mice. Results indicated a significant reduction in tumor growth in mice receiving the miR-218 overexpressing cells compared to the control group, suggesting that miR-218 acts as a tumor suppressor in NSCLC. Further analysis revealed decreased levels of RPTPα protein in the tumors harvested from the group of mice implanted with miR-218-overexpressing cells, which correlated with increased phosphorylation of c-Src at Tyr530 (tyrosine (Tyr) is located at 530 amino acid residue), indicating reduced c-Src activity. Notably, re-expressing RPTPα rescued tumor growth, while a mutated version of RPTPα (RPTPα^Y789F^) failed to show similar effects. These findings demonstrate that miR-218 inhibits tumorigenesis by targeting RPTPα and downregulating c-Src activity, highlighting its potential as a therapeutic target in lung cancer treatment strategies.

Another study by Zhu *et al*. evaluated the effects of miR-218 on tumor growth using nude mice^[73]^. Mice were implanted subcutaneously with human NSCLC H1975 cells, infected with a lentivirus for miR-218 overexpression or a control lentivirus. Following 1 week of implantation, it was found that tumors in the miR-218 overexpressing group exhibited significantly retarded growth compared to the control group. After 13 days, tumors were excised and analyzed for size and weight. Tumors from the miR-218 group were substantially smaller, confirming the tumor-suppressive effect of miR-218. Furthermore, miR-218-overexpressing tumors had higher levels of miR-218 and lower levels of EGFR, reinforcing the notion of direct inhibition of EGFR by miR-218. Immunohistochemical staining revealed reduced cell proliferation in miR-218 overexpressing tumors. Overall, the study demonstrated that miR-218 functions as a tumor suppressor in NSCLC by negatively regulating EGFR and could offer therapeutic potential for lung cancer treatment.

In a study, Shi *et al*. elucidated the role of miR-218 in NSCLC growth, utilizing two distinct *in vivo* experiments in BALB/c nude mice^[83]^. In the first experiment, researchers created stable cell lines in which H1299 cells overexpressed miR-218, while A549 cells had reduced expression. These cells were then injected subcutaneously into the flanks of the mice. After 22 days, the authors observed that miR-218-overexpressing tumors were significantly smaller compared to control tumors, while tumors with reduced miR-218 expression exhibited larger sizes and accelerated growth, suggesting that miR-218 inhibits tumor growth. Histological assessments showed reduced expression of Slug and ZEB2 in the smaller tumors, reinforcing the idea that miR-218 inhibits EMT. Meanwhile, the tumors harvested from reduced miR-218 expression had increased levels of Slug and ZEB2, which are markers of EMT. The second experiment aimed to investigate the effects of miR-218 on metastatic behavior. For that, tumor tissues from the first experiment were transplanted into the lungs of mice to analyze metastasis. The results indicated that mice harboring miR-218-overexpressing tumor cells exhibited significantly fewer metastatic lesions and less extensive invasion into lung tissue than controls. Histological staining again showed reduced levels of Slug and ZEB2 in these tumors, underscoring the role of miR-218 in inhibiting not only tumor growth but also metastatic spread. Overall, this study highlighted the critical function of miR-218 in inhibiting both tumor growth and metastatic spread in NSCLC, suggesting it as a potential therapeutic target.

Yang *et al*. conducted a study to evaluate the tumor-suppressive effect of miR-218 using A549 LUAD xenograft model in nude mice^[56]^. They utilized lentiviral vectors to stably express miR-218, or a control miRNA in A549 cells, which were then injected into the mice subcutaneously. The data demonstrated that tumors expressing miR-218 were significantly smaller than those in the control group, indicating the antitumor effects of miR-218. Furthermore, histological analyses of the tumors showed reduced Ki67 staining, signifying decreased cell proliferation, and lower levels of phosphorylated STAT3 were noticed in miR-218-expressing tumors. These findings confirmed that miR-218 inhibits activation of the IL-6/STAT3 signaling pathway.

Similarly, a study conducted by Li *et al*. assessed the effects of miR-218 on the growth and proliferation of a human LUAD model using female BALB/c nude mice^[79]^. To that end, A549 cells were administered subcutaneously into the right and left flanks of mice and once the tumors reached palpable size, a miR-218 inhibitor was injected into one flank, with a control miR sequence on the opposite flank, allowing for direct comparison. Through this approach, the study aimed to understand how inhibiting miR-218 affected tumor proliferation. After the treatment, the weights of the tumors were recorded and the results indicated that tumors treated with the miR-218 inhibitor exhibited a significantly greater volume and weight compared to the control group. This suggested that downregulation of miR-218 allowed for enhanced proliferation of malignant cells, indicating its role in suppressing tumor growth. The study highlighted the potential of miR-218 as a therapeutic target, demonstrating its regulatory role in inhibiting tumor growth and metastasis in LUAD, while paving the way for future miRNA-based cancer therapies.

Chen *et al*. aimed to assess the effects of miRNA-218–5p on radiation sensitivity in NSCLC using male BALB/c nude mice^[91]^. Researchers implanted radiation-resistant A549R cells and categorized the mice into control and experimental groups: miRNA-218–5p mimics, miRNA-negative control (miRNA-NC) + 12 Gy X-ray radiation, and miRNA-218–5p mimics + 12 Gy X-ray radiation. Following 2 days of treatment, tumor volumes and weight were measured. The data demonstrated that the mice receiving miRNA-218–5p mimics combined with X-ray radiation exhibited significantly smaller tumor volumes compared to the other groups, indicating enhanced radiosensitivity in combination with miR-218. Overall, the study suggests that targeting miRNA-218–5p could be a potential therapeutic strategy to improve radiotherapy outcomes in NSCLC, particularly for resistant cases, and highlights the promise of miRNA-based treatments in personalized cancer therapy.

While the preceding *in vivo* studies demonstrate that elevating miR-218 within tumor cells suppresses lung cancer growth and progression, the work by Yang *et al*. highlights a clear deviation from this trend^[119]^. In their xenograft model, maximal anti-tumor effects were achieved not by enhancing miR-218, but by inhibiting miR-218–5p in NK cells. Specifically, A549 cells were implanted subcutaneously into the nude mice, followed by treatment with IL-2-activated LNK cells transfected with either a miR-218–5p inhibitor or a negative control. Consistent with their mechanistic findings, suppression of miR-218–5p significantly reduced tumor growth, accompanied by increased SHMT1 expression in tumor tissues, indicating enhanced NK-cell activity *in vivo*. These results contrast with the majority of *in vivo* studies, in which upregulation of miR-218 inhibits tumor growth; in this case, downregulation of miR-218–5p in immune cells produced the anti-tumor effect. This discrepancy underscores that the *in vivo* role of miR-218 is context-dependent, varying across cellular compartments, and that therapeutic manipulation may have opposing consequences depending on whether the target is a tumor cell or an immune effector cell.

Across the *in vivo* studies assessed, several investigations of miR-218 in lung cancer relied on small, single-cohort sample sizes (in some cases, n < 10), which may limit the robustness and generalizability of the findings. Therefore, these results should be interpreted with caution and warrant further validation in larger, independent cohorts. In addition, the use of different mouse strains introduces important model-specific limitations that influence the interpretation of miR-218 functions in NSCLC. Nude mice, which lack functional T cells, permit efficient engraftment of human tumor cells but cannot model miRNA effects that depend on intact immunity^[120,121]^. BALB/c nude mice, although similarly immunodeficient, differ slightly in baseline tumor growth kinetics and inflammatory responses, which may alter microenvironmental influences on tumor progression^[122,123]^. SCID mice, with combined T- and B-cell deficiencies, provide an even more permissive setting for tumor establishment, yet their severely compromised immune surveillance restricts evaluation of miR-218’s potential immune-modulatory roles^[120,124]^. These inherent differences in immune competence complicate direct comparison across studies and underscore the importance of considering strain-specific limitations when interpreting *in vivo* miR-218 data.

### Evidence from clinical studies

Several studies have investigated the functional significance of miRNA-218 in NSCLC patients. In one study, Davidson *et al*. determined the expressions of miR-218 and its host genes, SLIT2 and SLIT3, in tumor samples and compared them to paired normal lung tissue^[58]^. For that, they collected lung tissue from 39 patients [18 SCC and 21 Adenocarcinoma (AC)] with or without smoking status. The analysis revealed that miR-218 was downregulated in 85% (33/39) of patients, and SLIT2 and SLIT3 downregulation was also observed in 97% (36/37) and 92% (34/37) of NSCLC tissues, respectively. Further analysis showed that miR-218 and SLIT2/3 downregulation were co-regulated as a concordant downregulation of miR-218 and SLIT2 was observed in 81% (30/37) of tissue samples. Moreover, miR-218 expression was found to be significantly reduced in the tissue samples of current smokers (14/39) and former smokers (19/39), and not in the samples of patients who had no history of smoking (6/39). These findings indicate a potential link of miR-218 expression with tobacco exposure, which is one of the major causes of lung cancer. The summaries of the clinical studies are given in Table 3 in the chronological order of their publication.

In another study, Wu *et al*. examined the association between miR-218 and PXN expressions and their correlation with tumor stages using tissue samples that were collected from 124 patients diagnosed with primary lung cancer^[74]^. The results showed that miR-218 levels were further downregulated in Stage III patients in comparison to Stage I and II patients. In contrast, the PXN mRNA and protein levels were found to positively correlate with tumor stages, suggesting that PXN expression in lung tumors is negatively associated with miR-218 expression. In addition, the high expression of miR-218 was also positively correlated with OS and RFS in lung cancer patients.

In addition, Zhu *et al*. conducted a study using NSCLC and normal adjacent tissues (NATs) from 6 patients, to assess the EGFR expression in lung cancer patients^[73]^. They found that EGFR mRNA and protein levels were significantly higher in NSCLC tissues in comparison to the NATs. The next study analyzed the expression of miR-218 in the same samples and found it to be negatively correlated with the EGFR expression, indicating that EGFR is a direct target of miR-218. These findings hold great significance considering the prevalence of EGFR mutations in NSCLC.

Shi *et al*. conducted a study to determine miR-218 expression in NSCLC across different TNM stages using tissue samples from 60 lung cancer patients^[83]^. The results showed that miR-218 expression was significantly downregulated in the NSCLC samples. Furthermore, the downregulation was inversely correlated with histological grade, and miR-218 levels were found to be drastically decreased in metastatic samples compared with non-metastatic NSCLC tissues. In addition, the researchers studied the expression levels of Slug/ZEB2 in the same tissues and found that they correlate negatively with the miR-218 expression, indicating miR-218’s potential against NSCLC metastasis.

In another study, Wang *et al*. studied the association of miR-218 with TRIM9 using 30 pairs of tumor tissues and adjacent non-tumor tissues collected from NSCLC patients^[80]^. The results revealed downregulation of miR-218 and upregulation of TRIM9 expression in NSCLC tissues. In addition, using immunohistochemical staining, the authors also confirmed increased levels of TRIM9 protein in NSCLC tissues. These findings suggested a negative association between TRIM9 and miR-218 in NSCLC patients.

Moreover, Chen *et al*. conducted a study to determine the effects of radiotherapy on miR-218 levels in NSCLC patients using blood samples collected from 42 patients before and after they received 60 Gy/30 min of radiotherapy^[91]^. The results showed that while the miR-218 levels were initially lower in the serum of patients, compared to healthy control, following radiotherapy, miR-218 expression increased significantly. In addition, they also tested the PRKDC levels in the same samples and found a negative correlation with the miR-218 levels. These findings indicate that miR-218 may target PRKDC and potentially enhance radiotherapy outcomes.

### Bioinformatics and large-cohort evidence

Multiple large-scale datasets and integrative bioinformatic analyses have independently validated the tumor-suppressive role of miR-218 in NSCLC. Early genome-wide mapping using array comparative genomic hybridization in 132 NSCLC tumors demonstrated that the host loci of miR-218, miR218–1 and miR218–2, reside within regions of recurrent copy-number loss, with matched expression profiling confirming significant downregulation of mature miR-218 in tumor tissues compared with adjacent normal lung^[58]^.

Subsequent bioinformatic studies further reinforce these observations. A multi-dataset integrative analysis identified FANCI (FA Complementation Group I) as a key gene inversely associated with miR-218 expression^[125]^. Notably, FANCI overexpression correlated with larger tumor size, lymph-node involvement, metastatic behavior, and poorer survival in LUAD^[125]^. The same study confirmed the miR-218-FANCI regulatory axis through TargetScan prediction and Gene Expression Omnibus (GEO)-based expression validation. Similarly, inverse correlations between miR-218 and oncogenic drivers such as CDCP1, RUNX2, PRKDC, and EGFR have been observed across public bioinformatics databases and tools such as The Cancer Genome Atlas (TCGA) and TargetScan^[73,77,90,91]^.

Clinical cohort-level evidence parallels these findings. In a prospective serum study of advanced NSCLC, 76.7% of patients exhibited miR-218–5p downregulation at diagnosis, with suppression persisting after multiple cycles of chemotherapy^[126]^. This circulating miRNA pattern mirrors the downregulation reported in tumor tissues and across public datasets^[126]^. Collectively, evidence from genomic copy-number mapping, multi-cohort bioinformatic analyses, and clinical plasma profiling converges to show that miR-218 is consistently and robustly downregulated across both major NSCLC subtypes, reinforcing its tumor-suppressive role in disease progression^[58,125,126]^.

## CONCLUSION AND FUTURE PERSPECTIVES

The role of miR-218 in the context of lung cancer has provided significant insights into its multifaceted roles as a tumor suppressor, prognostic indicator, and therapeutic target, offering a window into future research possibilities. However, the precise molecular mechanisms underlying these effects remain incompletely understood. For instance, while several miR-218 targets, such as Robo1, SLIT2, and pathways such as PI3K/AKT/mTOR and EMT regulators have been identified, their integrated network and specific roles require further elucidation. Furthermore, given that lower miR-218 expression is strongly associated with poor prognosis and decreased OS in NSCLC patients, therapeutic approaches aimed at restoring or enhancing miR-218 levels hold promise in reversing malignant phenotypes. This could include developing efficient miR-218 mimics, nanoparticle-based delivery systems, or viral vector therapy strategies to achieve targeted overexpression in tumor tissues while minimizing off-target effects^[127,128]^. However, significant challenges remain in optimizing the stability, specificity, and systemic delivery of such miRNA-based therapeutics^[129]^.

Another critical future direction is the evaluation of miR-218’s role in resistance to existing chemotherapeutic or targeted therapies. Investigating whether miR-218 modulation can sensitize tumor cells to conventional treatments could open new combinatorial therapeutic approaches for NSCLC management. In addition, integrating miR-218 expression profiling into existing diagnostic and prognostic models may enhance early detection strategies and treatment stratification for lung cancer patients. Future research should focus not only on validating these findings in large-scale, multicenter clinical cohorts but also on initiating early-phase clinical trials to assess the safety, pharmacodynamics, and efficacy of miR-218-targeted therapies in humans.

Beyond NSCLC, miR-218 may exhibit divergent roles in other lung cancer subtypes. In lung squamous cell carcinoma (LUSC), miR-218–5p expression progressively decreases from normal to tumor tissue, correlating with larger tumors, higher T stage, and enhanced migration and invasion, supporting a tumor-suppressive function in this context^[130,131]^. In SCLC, although data are limited, the lncRNA Linc00173 has been shown to act as a competing endogenous RNA (ceRNA) that sponges miR-218, reducing its inhibitory effect on targets such as Etk and thereby promoting proliferation and chemoresistance^[132]^. These observations highlight that miR-218’s function is context-dependent, influenced by tumor histology, transcriptional programs, and microenvironmental cues, and underscore the need for studies beyond NSCLC to fully elucidate its therapeutic potential across lung cancers.

In conclusion, although significant progress has been made in understanding the tumor-suppressive roles of miR-218, comprehensive mechanistic investigations and well-designed translational studies are essential to fully harness its potential as a biomarker and therapeutic target. In addition, challenges such as delivery stability, off-target effects, and clinical standardization must be addressed before therapeutic application can be realized. By bridging these gaps, miR-218-directed interventions could contribute meaningfully to improving the clinical management and outcomes of NSCLC patients.

## Figures and Tables

**Table 1.** Comparative summary of *in vitro* studies investigating miR-218 function in lung cancer

Mechanistic category	Targets	Comparative summary of miR-218 function in NSCLC cells	References
Apoptosis and cell-survival signaling	Bcl-2, BMI-1, YY1, PTEN, Mcl-1, Survivin, PARP, Caspase-3, Bax	Across these studies, miR-218 overexpression reduces the expression of multiple anti-apoptotic proteins (Bcl-2, BMI-1, Mcl-1, Survivin) while increasing pro-apoptotic markers (cleaved PARP, Caspase-3, Bax), leading to reduced cell viability and enhanced apoptosis. Conversely, loss of miR-218 has the opposite effect, supporting its consistent pro-apoptotic and tumor-suppressive roles	[64,72,75]
JAK-STAT proliferative signaling	IL-6R, JAK3	miR-218 decreases IL-6R and JAK3 levels, resulting in suppressed cytokine-driven proliferation. This links miR-218 to the inhibition of IL-6/STAT-type growth signaling in NSCLC	[56]
Growth-factor receptor signaling	EGFR	By targeting EGFR, miR-218–5p reduces receptor expression and downstream signaling, thereby suppressing cell migration and proliferation	[73]
Cell motility and structural support	PXN, RPTPa, CDCP1	PXN, RPTPa, and CDCP1 are proteins that promote cancer cell adhesion, motility and dissemination. Across these studies, miR-218 overexpression decreases their expression, leading to reduced migration, invasion and, in some models, diminished tumor growth and colony formation	[74,76–78]
EMT, invasion and metastasis	Robo1, Ecop, TRIM9, HMGB1, CDH2/N-cadherin, Slug, ZEB2, Robo1, CDCP1, HS3ST3B1	Multiple independent studies demonstrated that miR-218 downregulates EMT drivers at different levels, including surface receptors (Robo1, CDCP1, HS3ST3B1), transcriptional regulators (Slug, ZEB2), mesenchymal markers (CDH2) and pro-invasive factors (Ecop, HMGB1, TRIM9). Across these targets, miR-218 consistently reduces EMT marker expression as well as cell migration and invasion, indicating that inhibition of EMT and metastatic potential is a central function of miR-218 in NSCLC	[76,77,79–85]
Transcriptional growth control	MEF2D	By suppressing MEF2D, miR-218 inhibits lung cancer cell growth, supporting its broader role in limiting cancer progression	[86]
Metabolic regulation and stress response	SHMT1, ERO1A, GLUT1	miR-218 affects several metabolic pathways: it reduces GLUT1-mediated glucose uptake, interferes with folate/one-carbon metabolism through SHMT1, and disrupts oxidative and ER stress responses via ERO1A. Across these pathways, miR-218 consistently limits metabolic activity, reduces tumor growth, and suppresses angiogenesis	[87–89]
Chemotherapy sensitivity (platinum agents)	RUNX2, Bcl-2/BMI-1/Mcl-1/Survivin axis	miR-218 improves the response to cisplatin by downregulating RUNX2 and enhances carboplatin-induced cell death by reducing survival-related proteins. Overall, miR-218 acts as a sensitizer to platinum-based drugs by weakening resistance pathways	[64,72,75,90]
Targeted therapy resistance (EGFR-TKI)	HOXA1	In gefitinib-resistant NSCLC, restoration of miR-218 levels decreases HOXA1 levels, reducing proliferation and promoting gefitinib-induced apoptosis, indicating that loss of miR-218 contributes to resistance to EGFR-TKIs	[65]
Radiation response and DNA-damage repair	PRKDC	miR-218 directly suppresses PRKDC, a key DNA repair enzyme, leading to increased unrepaired DNA damage, enhanced apoptosis, and greater radiosensitivity, including in radiation-resistant models	[91]

Bcl-2: B-cell lymphoma; BMI-1: B lymphoma Mo-MLV insertion region 1 homolog; YY1: Yin Yang 1; PTEN: phosphatase and tensin homolog; Mcl-1: myeloid cell leukemia sequence 1; PARP: poly-ADP ribose polymerase; JAK: Janus kinase; STAT: signal transducer and activator of transcription; IL-6 - Interleukin-6; EGFR: epidermal growth factor receptor; PXN: paxillin; RPTPα: receptor protein tyrosine phosphatase alpha; CDCP1: CUB-domain-containing protein 1; Robo1: roundabout homolog 1; Ecop: EGFR-coamplified and -overexpressed protein; TRIM9: tripartite motif 9; CDH2: N(Neural)-cadherin; ZEB2: Zinc finger E-box-binding homeobox 2; HS3ST3B1: Heparan sulfate D-glucosamine 3-O-sulfotransferase 3B1; MEF2D: myocyte enhancer factor 2D; SHMT1: serine hydroxymethyltransferase 1; ERO1A: endoplasmic reticulum oxidoreductase 1 alpha; GLUT1: glucose transporter 1; RUNX2: Runt-related transcription factor 2; HOXA1: homeobox A1; PRKDC: protein kinase, DNA-activated, catalytic subunit; NSCLC: non-small-cell lung cancer; EMT: epithelial-mesenchymal transition; IL-6R: interleukin-6 receptor; EGFR-TKI: EGFR-tyrosine kinase inhibitor.

**Table 2.** Summary of the *in vivo* studies defining the role of miR-218 in lung cancer

Model/Experimental design	Treatment groups	Timeline/Endpoint	Key findings	References
Intracardiac injection of metastatic NSCLC cells with tetracycline-inducible pri-miR-218; SCID mice	Doxycycline vs. control; pri-miR-218 ON/OFF	Metastasis was monitored at approximately 4 weeks; and survival was followed until endpoints	miR-218 overexpression reduced metastatic burden and improved survival	[76]
Subcutaneous xenografts using A549 cells with RPTPα mutation ± miR-218 modulation; BALB/c nude mice (n = 4/group)	A549-RPTPα mutation ± miR-218 manipulation	Tumor growth was monitored every 2 days starting at week 2, with the endpoint at approximately 5 weeks	miR-218 suppressed RPTPα expression and inhibited tumorigenesis	[78]
Subcutaneous xenografts with H1975 cells stably overexpressing miR-218; nude mice (n = 10/group)	miR-218 mimic vs. control	Tumors were measured on days 7, 9, 11, and 13; and the animals were sacrificed thereafter	miR-218 overexpression suppressed NSCLC xenograft growth	[73]
H1299 and A549 cell lines were injected into the posterior flanks; BALB/c nude mice (n = 6)	miR-218 overexpression vs. NC; anti-miR-218 vs. anti-NC	Tumors were measured every 2 days after Day 12, and the animals were sacrificed after Day 22	Overexpression of miR-218 suppressed Slug and ZEB2 expression and inhibited tumor growth and metastasis	[83]
Orthotopic lung implantation of tumor fragments from subcutaneous xenografts; BALB/c nude mice	miR-218 overexpression *vs*. control	Tumor growth and metastasis were assessed 7 weeks post-implantation		
Subcutaneous A549 xenografts; BALB/c nude mice (n = 5/group)	miR-218-LV vs. control-LV	Tumor volume was measured weekly, and the animals were sacrificed at week 5 post-injection	miR-218 overexpression inhibited tumor growth by targeting STAT3	[56]
Subcutaneous xenografts using A549 cells; 10 BALB/c nude mice (n = 8)	miR-218 inhibitor *vs*. control	Tumor volume was measured every other day after reaching 50mm^3^, with the endpoint at 30 days	miR-218 inhibition promoted NSCLC xenograft growth	[79]
Subcutaneous A549R xenografts; BALB/c nude mice (n = 3/group)	miRNA-NC, miR-218 mimic; each ± 12 Gy X-ray	Tumor volume was measured every 3 days, with the endpoint at 25 days	miR-218–5p increased radiosensitivity *in vivo*	[91]

NSCLC: Non-small-cell lung cancer; RPTPα: receptor protein tyrosine phosphatase alpha; ZEB2: zinc finger E-box-binding homeobox 2; STAT3: signal transducer and activator of transcription 3; SCID: severe combined immunodeficient; NC: negative control; LV: lentivirus.

**Table 3.** Summary of clinical studies defining the role of miR-218 in lung cancer

Study design	Patient characteristics/sample size	miR-218 expression levels	Findings	References
To study the expression of the host genes SLIT2 and SLIT3, and miR-218 in NSCLC patient samples	Samples were collected from 39 lung cancer patients	miR-218 expression was downregulated by 4-fold in NSCLC samples	SLIT2/3 and mir-218 expressions were significantly downregulated in NSCLC samples. Furthermore, smokers (current and former) had significantly decreased levels of miR-218 expression	[58]
To determine the association of PXN with miR-218 expression	Samples were collected from 124 patients with primary lung cancer	miR-218 levels decreased as lung cancer progressed through its stages	PXN expression was significantly elevated and miR-218 expression was downregulated in tumor samples relative to normal tissues	[74]
To determine whether EGFR expression is associated with miR-218 expression	Samples were collected from six lung cancer patients	miR-218 expression was nearly 2-fold higher in normal lung samples compared to NSCLC tissues	EGFR mRNA and protein levels were consistently higher in tumor samples, relative to normal tissues; miR-218 expression was downregulated	[73]
To determine the association of Slug/ZEB2 with miR-218 expression	Samples were collected from 60 NSCLC patients	miR-218 levels were downregulated in NSCLC and declined significantly with advancing TNM stages	Slug/ZEB2 expression was markedly elevated and miR-218 expression was downregulated in tumor samples relative to normal tissues	[83]
To determine the association of TRIM9 with miR-218 expression	Samples were collected from 50 NSCLC patients who did not receive any prior treatments	miR-218 expression was downregulated by 2-fold in NSCLC samples	TRIM9 expression was markedly higher and miR-218 expression was downregulated in tumor samples relative to normal tissues	[80]
To determine whether miR-218 synergistically enhances the efficacy of radiotherapy	Samples were collected from 42 patients with NSCLC who received radiotherapy	miR-218 levels were nearly 2-fold higher in the serum of control patients compared to lung carcinoma patients	miR-218 level in the serum of LC patients after radiotherapy was evidently higher than pre-radiotherapy. PRKDC expression was negatively correlated with miRNA-218–5p levels in the serum of LC patients	[91]

SLIT2/3: Slit guidance ligand 2/3; NSCLC: non-small-cell lung cancer; PXN: paxillin; EGFR: epidermal growth factor receptor; ZEB2: zinc finger E-box-binding homeobox 2; TNM: tumor, node, metastasis; PRKDC: protein kinase, DNA-activated, catalytic subunit; LC: lung cancer.
